# Did female prisoners with mental disorders receive psychiatric treatment before imprisonment?

**DOI:** 10.1186/s12888-015-0387-z

**Published:** 2015-01-22

**Authors:** Adrian P Mundt, Sinja Kastner, Jan Mir, Stefan Priebe

**Affiliations:** 1 Unit for Social and Community Psychiatry (WHO Collaborating Centre for Mental Health Services Development), Queen Mary University of London, Newham Centre for Mental Health, London, E13 8SP UK; 2 Escuela de Medicina sede Puerto Montt, Universidad San Sebastián, Puerto Montt, Chile; 3 Department of Psychiatry and Psychotherapy, Charité Universitätsmedizin Berlin, Charité Campus Mitte, Charitéplatz 1, 10117 Berlin, Germany

**Keywords:** Prisoners, Mental health, Psychiatric hospitalized care, Outpatient mental health treatment

## Abstract

**Background:**

Throughout the world, high prevalence rates of mental disorders have been found in prison populations, especially in females. It has been suggested that these populations do not access psychiatric treatment. The aim of this study was to establish rates of psychiatric in- and outpatient treatments prior to imprisonment in female prisoners and to explore reasons for discontinuation of such treatments.

**Methods:**

150 consecutively admitted female prisoners were interviewed in Berlin, Germany. Socio-demographic characteristics, mental disorders, and previous psychiatric in- and outpatient treatments were assessed by trained researchers. Open questions were used to explore reasons for ending previous psychiatric treatment.

**Results:**

A vast majority of 99 prisoners (66%; 95% CI: 58–73) of the total sample reported that they had previously been in psychiatric treatment, 80 (53%; 95 CI: 45–61) in inpatient treatment, 62 (41%; 95 CI: 34–49) in outpatient treatment and 42 (29%; 21–39) in both in- and outpatient treatments. All prisoners with psychosis and 72% of the ones with any lifetime mental health disorder had been in previous treatment. The number of inpatient treatments and imprisonments were positively correlated (rho = 0.27; p < 0.01). Inpatient treatment was described as successfully completed by 56% (N = 41) of those having given reasons for ending such treatment, whilst various reasons were reported for prematurely ending outpatient treatments.

**Conclusion:**

The data do not support the notion of a general ‘mental health treatment gap’ in female prisoners. Although inpatient care is often successfully completed, repeated inpatient treatments are not linked with fewer imprisonments. Improved transition from inpatient to outpatient treatment and services that engage female prisoners to sustained outpatient treatments are needed.

## Background

Female prisoners were estimated to have high rates of mental health and substance use disorders. Prevalence rates of severe mental disorders were estimated to be 3.9% for psychoses and 14.1% for major depression in a recent meta-analysis [1]. Prevalence estimates ranged between 18% and 24% for alcohol use and between 30% and 60% for illicit drug use disorders [2]. Moreover, 42% of female prisoners were estimated to have a personality disorders [3]. Most studies on prison inmates so far included predominantly or completely male samples [4], despite evidence that female prisoners may have higher prevalence rates of mental disorders than men [3,5-9]. Reincarceration rates are higher for people with severe mental health problems [10], especially when there are comorbid substance use disorders [11]. It has been suggested that people with severe mental disorders were increasingly involved with the penal justice system, as they may have reduced access to psychiatric care [12-14].

Psychiatric bed reductions may be associated with increasing prison populations [15]. To further understand this relationship, it is necessary to evaluate whether the same people are admitted to both institutions. Psychiatric treatment histories of prisoners may clarify the proportion of prisoners with mental health problems that at times is served in psychiatric hospitals. So far, only few studies reported psychiatric treatment histories of prisoners, none as primary outcome. Therefore, we searched the literature on prevalence rates of mental disorders in prison populations [1]. In those studies, estimations for the rate of female prisoners with psychiatric treatment prior to incarceration ranged from 36% to 75% [16-20]. Studies from Northern Europe with small numbers had pointed to high rates. The rates for having been not only in psychiatric treatment but specifically in inpatient services prior to imprisonment ranged from 8% [21] to 30% [19] in female prisoners.

It is necessary to further assess and specify psychiatric in- and outpatient treatment histories of female prisoners, as previous studies are still inconclusive and inconsistent. Further understanding of the degree of interdependence between the psychiatric and penal justice systems is useful for service development. Acknowledging reasons for ending or abandoning previous treatments could provide a starting point to improve the engagement in mental health treatment of women with penal justice involvement. The present study explored the psychiatric in- and outpatient treatment histories of female prisoners in Berlin, Germany and reasons for having ended previous psychiatric treatments.

## Methods

This was a cross-sectional study of a sample of consecutively admitted female prisoners in Berlin, Germany.

### Sample

The sample was recruited from all consecutive female committals to the penal justice system in Berlin, including the open, semi-open and the closed systems. The sample did not include women regarded to have reduced legal responsibility due to mental disorders in terms of §20 or §21 of the German Criminal Law. We aimed to recruit a total sample of 150 participants. The sample size was exploratory and expected to yield percentage estimates with reasonable 95% confidence intervals (CI) for the total sample, i.e. 10% (95% CI: 5–15) or 20% (95% CI: 14–26). Prisoners with all types of verdict such as people in detention, remand prisoners and convicted prisoners were included in the study. The interview was usually scheduled within a week after imprisonment and always within the first month of imprisonment. Exclusion criteria for the study were the inability to communicate in German and a lack of capacity to provide informed consent.

### Measures

Age, marital and employment status, educational and income level, were assessed on structured questions. The variables were dichotomized as living alone or with partner, education as low (comprising the categories 0–2 of the International Standard Classification of Education [ISCED] with all levels of education up to lower secondary levels of education) and high educational level (comprising the categories 3–6 of the ISCED with all educational levels from upper secondary level and higher [22]). Employment status was dichotomized to employed (including people in training under the age of 28 years) and unemployed (including people in training of 28 years or older and retired people). This classification is in accordance with German legislation which requires the long term unemployed to take part in trainings to continuously qualify for social benefits [23]. The income level was dichotomized to € < 990 and € ≥ 990 per month, which was the line of relative poverty for a single person household in 2010 (http://www.diw.de/de/diw_01.c.411565.de/presse/diw_glossar/armut.html). The type of criminal offense was assessed.

The fully structured Mini International Neuropsychiatric Interview (MINI) 6.0 [German version] was conducted to assess mental health and substance use disorders. The MINI was developed by Sheehan and Lecrubier [24] to categorize a part of the axis I mental disorders and antisocial personality disorders according to the fourth version of the Diagnostic and Statistical Manual of Mental Disorders (DSM-IV). The interview schedule was supplemented by the module for borderline personality disorder of the Structured Clinical Interview for DSM-IV (SCID) [25]. For the purpose of reporting treatment histories, diagnoses were then grouped into the following categories: ‘any disorder’ including all disorders covered in the MINI and Borderline disorder, screened for separately; ‘affective disorders’ including major depression, recurrent major depression, bipolar disorders, dysthymia and affective psychosis; ‘anxiety disorders’ including panic disorder, agoraphobia, social phobia, post-traumatic stress disorder, obsessive-compulsive disorder and generalized anxiety disorders; ‘eating disorders including anorexia’, bulimia also covered by the MINI was not found; and ‘psychotic disorders’ including probable non-affective psychoses; and the two personality disorders antisocial and borderline personality disorders were grouped together.

Previous imprisonment as remand or sentenced prisoner, psychiatric and psychotherapeutic treatment history were assessed. From here on, the term psychiatric is used comprising ‘psychiatric and psychotherapeutic’ because in this context they are referred to synonymously. History of previous imprisonments and treatments were based on subjective recall and not corroborated by objective administrative data. For the mental health treatment histories, recall was believed to be superior to objective health records that may have a varying quality as other clinical routine data. With respect to the previous imprisonments this was a pragmatic decision due to confidentiality concerns on the side of the prison administration to extract data from penal justice records. The decision was based on the rationale that recall with respect to previous imprisonments was sufficiently accurate in the study population. For cases of previous imprisonments in other states or countries and prior to the introduction of electronic records, subjective recall may be more accurate than electronic files or criminal records. Reasons for ending in- and outpatient care were assessed using the following open questions: ‘What were the reasons for ending psychiatric in-patient treatment?’ and ‘What were the reasons for ending psychiatric/psychotherapeutic outpatient treatment?’ Those questions were followed up by using a specification, if the treatment was prematurely ended on either side or abandoned: ‘What were the reasons for that?’

### Procedure

The capacity to give informed consent was tested by assessing the potential participant’s ability to understand the purpose of the study. The field team consisted of two clinical psychologists trained and supervised by a senior consultant psychiatrist in using the instruments. The interviews lasted for 45–60 minutes and were held in a separate room of the prison to ensure confidentiality. The data were collected between April 2012 and May 2013. All interviewees provided written informed consent. The study was approved by the Ethics Board of the Charité Universitätsmedizin Berlin (EA1/302/11) and by the legal justice department of the State of Berlin, Germany (reference AL, 20.01.2012).

### Analyses

Socio-demographic characteristics and prevalence rates of mental disorders were calculated as per cent values with 95% CI using a bootstrap algorithm for the groups with previous psychiatric treatment and without previous psychiatric treatment. Prevalence rates of mental disorders for the total sample will be reported in a forthcoming paper. Two-sided Spearman’s correlations for non-parametric tests were used to explore correlations between the number of previous imprisonments and the following: having been in any psychiatric treatment, in previous inpatient treatment and in previous outpatient treatment. To test the relationship between having been in psychiatric inpatient treatment with the number of previous imprisonments found in bivariate analyses, we conducted a Poisson generalized linear regression analysis with previous imprisonments as the dependent variable. We introduced having been in any treatment, having been in inpatient treatment and having been in outpatient treatment as independent variables.

To identify high users of both systems, a group of prisoners with ≥2 previous admissions to psychiatric inpatient treatments and ≥2 previous imprisonments were identified. Values of p < 0.05 were considered statistically significant. The statistical analyses were made using SPSS version 20.0 and Stata 12.0.

The answers to the open questions were subjected to content analysis [26]. We used a conventional approach to content analysis. Codes were derived from the data and defined during data analysis. Two of the authors independently coded the data.

## Results

### Recruitment

During the recruitment period, 338 women entered the central facility for the admission of female prisoners to the penal justice system in Berlin. Sixty women were transferred to other detention centres within days or were imprisoned for only a few days and, therefore, could not be approached for inclusion to the study. Of the remaining 278 women, 198 fulfilled the inclusion criteria, while 80 women did not: 69 were not able to speak sufficient German, and 11 had severe cognitive or psychological incapacities and were not able to give informed consent to participate in the study. Of the 198 fulfilling the inclusion criteria, 48 declined participation, and 150 agreed to participate.

### Psychiatric treatment trajectories

Table 1 reports the types of psychiatric treatment received for the whole sample.

**Table 1:** **Prevalence rates and 95%-confidence intervals for the psychiatric treatment history in a sample of consecutively admitted female prisoners**

Psychiatric treatment history	N	%	95% CI
Any psychiatric service	99	66	58-73
Never used any psychiatric service	51	34	26-41
Inpatient psychiatric service	80	53	45-61
Only inpatient psychiatric service	37	25	18-32
Outpatient psychiatric service	62	41	34-49
Only outpatient psychiatric service	19	13	7-18
Both inpatient and outpatient psychiatric service	43	29	21-39

Two thirds (n = 99; 66%; 95% CI: 58–73) of the interviewees had previously received some form of psychiatric treatment. About half of the sample (n = 80; 53%; 95% CI: 45–61) had been in inpatient treatment; 62 prisoners (41%; 95% CI: 34–49) had received psychiatric outpatient treatment; and 43 prisoners (29%; 95% CI: 21–39) had received both in- and outpatient psychiatric treatment; 37 prisoners (25%; 95% CI: 18–32) had been hospitalized without ever having used any outpatient psychiatric service; and 19 (13%, 95% CI: 7–18) had been treated in outpatient psychiatric services, without ever having been in psychiatric inpatient care.

Previously hospitalized individuals (n = 80) had a mean number of 3.7 (95% CI: 2.7-4.7) psychiatric inpatient treatments. The number of previous imprisonments was positively correlated with having been in any psychiatric treatment (Spearman’s rho = 0.23; p < 0.01) and also with the number of previous psychiatric inpatient treatments (Spearman’s rho = 0.27; p < 0.01), but not with previous outpatient treatments (Spearman’s rho = 0.01; p = 0.88). The generalized linear model with the number of previous imprisonments as dependent variable is shown in Table 2. Having been in psychiatric inpatient treatment associated with the number of previous imprisonments (incidence risk ratio of 2.49; p = 0.005). Previous outpatient treatment and any treatment did not show a significant relationship with the number of previous imprisonments.

**Table 2:** **Poisson generalized linear regression model with number of previous imprisonments as dependent variable**

Independent variable	Incidence rate ratio	95% confidence interval	p-value
Inpatient treatment	2.49	1.31-4.73	.005
Outpatient treatment	0.81	0.57-1.14	.23
Any treatment	0.81	0.39-1.79	.58

Twenty-nine participants had at least two previous psychiatric inpatient treatments and had been admitted at least for the second time to the penal justice system (n = 29; 19% of the total sample; 95% CI: 13–27). Only 15 out of these 29 prisoners (52%) had received outpatient treatment.

### Socio-demographic characteristics

Socio-demographic characteristics are shown for the groups with and without previous psychiatric treatment (Table 3).

**Table 3:** **Socio-demographic characteristics of consecutively admitted female prisoners with and without history of previous psychiatric treatment**

Socio-demographic characteristic	Total sample			No previous psychiatric treatment history			Previous psychiatric treatment		
	N = 150	Mean	95% CI	N = 51	Mean	95% CI	N = 99	Mean	95% CI
Age		34	33-36		36	33-39		34	31-36
Previous imprisonments		1.2	0.9-1.5		0.9	0.5-1.4		1.4	1.1-1.8
		**%**			**%**			**%**	
Living alone	139	93	88-97	43	84	74-94	96	97	93-100
Co-residing	11	7	3-12	8	16	6-26	3	44	0-7
**Educational level**									
ISCED 0-2	89	59	51-67	29	57	43-71	60	61	51-71
ISCED 3-6	61	41	33-49	22	43	30-58	39	39	29-49
**Employment**									
Unemployed	113	75	68-82	36	71	58-83	77	78	69-86
Employed	37	25	18-32	15	29	17-42	22	22	14-31
**Income level**									
Below poverty line	119	79	73-86	40	78	67-90	79	80	72-87
Above poverty line	31	21	14-27	11	22	14-38	20	20	15-32
**Family situation**									
Children	104	69	62-77	37	73	59-84	67	68	58-77
No children	46	31	23-38	14	28	15-40	32	32	24-42
**Offense category**									
Failure to pay a fine	69	46	39-54	21	41	27-55	48	49	38-58
Theft/fraud	35	23	17-30	13	26	14-38	22	22	14-31
Remand prisoners	16	11	6-16	8	16	6-26	8	8	3-14
Violent crimes	15	10	5-15	3	6	0-13	12	12	6-19
Related to drugs	10	7	3-11	4	8	2-16	6	6	2-12
Related to immigration	6	4	1-7	3	6	0-13	3	3	0-7

Most of the participants in either group had been living alone, had low educational levels, were unemployed prior to admission, had incomes below the poverty line and had committed minor non-violent offenses such as not paying a fine, theft or fraud.

### Treatment histories for specific diagnostic groups

Table 4 shows by diagnostic group whether they had received previous psychiatric treatment. One hundred and fifteen prisoners (77%) of the sample had at least one current and 136 prisoners (91%) at least one lifetime disorder.

**Table 4:** **Previous psychiatric treatment histories for different diagnostic groups**

	Total sample		No previous treatment		Previous psychiatric treatment		Previous inpatient treatment		Previous outpatient treatment	
Mental disorder	N = 150	%	N = 51	%	N = 99	%	N = 80	%	N = 62	%
At least one **current** disorder	115	77	29	25	86	75	72	63	51	44
At least one **lifetime** disorder	136	91	38	28	98	72	80	59	61	45
**Current** affective disorder^1^	35	23	7	20	28	80	22	63	22	63
**Lifetime** affective disorder	97	65	22	23	75	77	60	62	54	56
Substance-related disorder **(one year)**	93	62	21	23	72	77	64	69	41	44
**Current** anxiety disorder^2^	55	37	11	20	44	80	37	67	30	55
**Lifetime** anxiety disorder	65	43	14	22	51	78	43	66	35	54
Eating Disorder: Anorexia	4	3	0	0	4	100	3	75	3	75
**Current** psychotic disorder	3	2	0	0	3	100	3	100	2	67
**Lifetime** psychotic disorder	8	5	0	0	8	100	7	88	5	63
Borderline or antisocial personality disorder	53	35	10	19	43	81	41	77	28	53

^1^including major depression, recurrent major depression, bipolar disorders, dysthymia and affective psychosis.

^2^including panic disorder, agoraphobia, social phobia, post-traumatic stress disorder, obsessive-compulsive disorder and generalized anxiety disorders.

All prisoners with psychotic disorders and eating disorders had received psychiatric treatment. For the other diagnostic groups the rate of previous treatment was close to 80%. Close to 20% with most diagnoses had never been in psychiatric treatment. Irrespective of the diagnostic group, rates of previous inpatient treatment were equal or higher than rates of previous outpatient treatment.

### Reasons for ending previous psychiatric treatments

Seventy-three out of 80 prisoners with previous inpatient treatments reported a total of 78 reasons for having ended psychiatric inpatient treatments. By far the most commonly reported reason was that the treatment had been effective and that the mental health problem had improved because of it (n = 41; 56%). Detoxification treatments were described as successful when they had led to at least temporary abstinence or stable substitution. These treatments were perceived as not successful when they were abandoned during hospitalization. Treatments were discontinued mostly for drug craving (n = 13; 18%), problems with the setting such as sharing the ward with ‘mad’ people and not liking psychiatry in general (n = 7; 10%), dismissal for conduct problems (n = 7; 10%), and the experience of coercive measures (n = 6; 8%). Less frequent were administrative problems (n = 3; 4%) or the end of a forensic treatment verdict (n = 1; 1%).

Forty-five out of 62 prisoners with previous outpatient treatments reported a total of 53 reasons for having ended outpatient treatments. Only 16 (36%) stated that the treatment had been successfully completed with an improvement of the given mental health problem. Patients discontinued outpatient treatment because they felt unable to talk (n = 12; 27%), relapsed into substance abuse (n = 6; 13%), had no access to further treatment (n = 4; 9%) or were imprisoned during treatment (n = 4; 9%).

## Discussion

### Main findings

Female prisoners report very high rates of access to psychiatric treatment, especially to inpatient treatment. About 20% of prisoners with mental disorders across the most common diagnostic categories have reported to have never been in psychiatric treatment. However, the vast majority have received psychiatric treatment, and many even repeatedly. This was particularly in inpatients settings where most participants felt the treatment was successful.

### Strengths and limitations

The study recruited consecutively admitted women so that the sample included female prisoners regardless of their length or type of verdict. Diagnoses were established by independent researchers, using standardised instruments.

Yet, the study also has several limitations. Firstly, the recruitment was carried out in one single site that receives all female prisoners in Berlin and it is debatable to what degree the findings can be generalised to larger parts of Germany or other European contexts. Secondly, the sample did not include prisoners whose German language proficiency was insufficient to understand the interview questions. However, we included non-native speaking immigrants with sufficient language proficiency. Thirdly, we did not include people without capacity to give informed consent such as severe cognitive impairments or acute agitation. And finally, the data on the treatment history and the history of previous imprisonments were based on the recall of the participants and not corroborated by objective data.

### Comparison against the literature

In the following, the findings are first discussed against a study reporting treatment histories for male prisoners in Germany and then compared with reports from other countries with different legal and social contexts. In Germany, 31% of convicted male prisoners had been previously admitted to inpatient psychiatric treatment as compared to 53% of the females in this study and 20% of the male prisoners had previously used outpatient care as compared to the 41% of the females in our sample [27]. Our findings indicate that female prisoners have higher rates of previous psychiatric inpatient and outpatient treatment than male prisoners in Germany.

Female prisoners in New Zealand had rates of previous psychiatric hospitalizations of 8% [21], much lower than in our study and than in other studies from Europe. From Ireland lower rates of previous mental health treatment (34% including inpatient and outpatient treatments) in female committals to prison were reported as compared to psychiatric treatment histories of female prisoners in Berlin in this study (66%) [16]. A study from England and Wales reported 12% inpatient and 24% outpatient treatment histories as adults and 9% child guidance in sentenced female prisoners [17]. The highest rates of psychiatric treatment history reported so far for female prisoners were from a small sample n = 33 in Finland indicating 75% of previous psychiatric treatment and 30% of psychiatric hospitalization [19]. As compared to our study, the rates were higher for any psychiatric treatment but lower for the inpatient treatment.

Not only in Germany but also internationally, the rates were lower for male prisoners as compared to females [17]. As for the female prisoners, the access to psychiatric care, especially in the inpatient sector prior to imprisonment may be higher in Germany for male prisoners as compared to other countries, such as the US [28], Ireland [29] or Australia [30]. It could be a characteristic of the German legal context and the reimbursement system in health care that inpatient treatment is more ubiquitous and accessible for this population than outpatient care. The rate of psychiatric hospital beds is high in Germany and people with addiction are accepted for acute inpatient detoxification [31]. However, the high rate of previous inpatient treatment cannot only be attributed to one specific diagnostic group such as addiction. The finding that having been hospitalised in psychiatry significantly correlated with the number of previous imprisonments is consistent with the hypothesis that people with severe mental disorders are at high risk of re-offending [10] and for the direct interdependence of the penal justice system and the psychiatric inpatient services [15]. The repeat hospitalizations in psychiatry are an indicator for the severity and the chronicity of the disorders that come together with repeat penal justice involvement, which could mean that the crimes of this group tend to be minor, that rehabilitation usually fails and that the execution of punishment fails to disincentive further criminal behaviour. This finding could also indicate that the psychiatric treatment as currently provided is ineffective in reducing the rate of subsequent imprisonment, otherwise an inverse relationship would have been expected. A history of psychiatric treatment including inpatient treatment that was perceived as successful appears not to prevent imprisonment. Prospective studies are necessary to confirm this.

## Conclusions

The data do not support the notion of a general ‘mental health treatment gap’ in female prisoners. The number of prisoners with all types of mental health problems who had not received psychiatric treatment is much smaller than the number of those who had received such treatment and smaller than expected from the literature. More research is required in different national, legal and social contexts exploring exact pathways to psychiatric care of people with penal justice involvement. Better treatments that engage people into sustained outpatient care and reduce re-incarceration are needed for female prisoners.
